# Effect of Obesity on Masticatory Muscle Activity and Rhythmic Jaw Movements Evoked by Electrical Stimulation of Different Cortical Masticatory Areas

**DOI:** 10.3390/jcm12113856

**Published:** 2023-06-05

**Authors:** Ruixin Li, Chiho Kato, Akiyo Fujita, Yasunori Abe, Takuya Ogawa, Hideyuki Ishidori, Eri Misawa, Hidemasa Okihara, Satoshi Kokai, Takashi Ono

**Affiliations:** Department of Orthodontic Science, Graduate School of Medical and Dental Sciences, Tokyo Medical and Dental University (TMDU), 1-5-45 Yushima, Bunkyo-ku, Tokyo 1138510, Japan

**Keywords:** obesity, rhythmic jaw movements, cortical mastication area, intracortical micro-stimulation, electromyography, digastric muscle, Zucker rat

## Abstract

This study investigates rhythmic jaw movement (RJM) patterns and masticatory muscle activities during electrical stimulation in two cortical masticatory areas in obese male Zucker rats (OZRs), compared to their counterparts—lean male Zucker rats (LZRs) (seven each). At the age of 10 weeks, electromyographic (EMG) activity of the right anterior digastric muscle (RAD) and masseter muscles, and RJMs were recorded during repetitive intracortical micro-stimulation in the left anterior and posterior parts of the cortical masticatory area (A-area and P-area, respectively). Only P-area-elicited RJMs, which showed a more lateral shift and slower jaw-opening pattern than A-area-elicited RJMs, were affected by obesity. During P-area stimulation, the jaw-opening duration was significantly shorter (*p* < 0.01) in OZRs (24.3 ms) than LZRs (27.9 ms), the jaw-opening speed was significantly faster (*p* < 0.05) in OZRs (67.5 mm/s) than LZRs (50.8 mm/s), and the RAD EMG duration was significantly shorter (*p* < 0.01) in OZRs (5.2 ms) than LZR (6.9 ms). The two groups had no significant difference in the EMG peak-to-peak amplitude and EMG frequency parameters. This study shows that obesity affects the coordinated movement of masticatory components during cortical stimulation. While other factors may be involved, functional change in digastric muscle is partly involved in the mechanism.

## 1. Introduction

Since 1975, the global prevalence of obesity has increased drastically [1]. The World Health Organization reported that about 13% of the world’s adult population (11% of men and 15% of women) were obese in 2016, and almost half of the children under five who were overweight or obese in 2019 lived in Asia. Obesity is a highly heritable trait with 50–90% of heritability estimates [2,3].

Obesity leads to poor skeletal muscle quality. In terms of muscle contractile ability, obesity is associated with a greater absolute force in weight-bearing muscles because of the greater load that the muscles experience; however, when normalized to body mass, the muscle performance of obese individuals is significantly lower than that of lean individuals [4,5,6,7]. As for the muscle fiber type, obesity promotes the shift in fiber types from “slow” to “fast” through various pathways, such as AMPK and Ca^2+^ signaling in skeletal muscles [8,9]. Moreover, obesity is also associated with decreased fatigue resistance during isometric endurance exercises [10]. 

Obesity is related to changes in oral structure and function. As for the tongue, previous studies have revealed more fat infiltration in the tongue muscles of genetically obese Zucker rats (OZRs) and rats fed a high-fat diet compared to that in lean Zucker rats (LZRs) [11,12]; however, in situ, force production of the tongue muscle remained unchanged between obese and lean rats [13]. Previous studies also reported smaller airway caliber with greater upper airway soft tissue structure volume in obese mouse models [14]. Obesity is also a risk factor for obstructive sleep apnea [15]; therefore, it is important to investigate if these organic changes caused by obesity in oral tissues lead to motor change. Its results may give insights into clinical problems regarding obesity. Obesity is also associated with altered masticatory behaviors, such as a faster eating rate [16,17,18], fewer chewing cycles [18,19], and lower chewing degrees [20]. Studies on the effect of obesity on masticatory muscles are limited, although altered masticatory behaviors are considered an important reason for obesity [21]. A previous study has reported differences in the longitudinal and transverse sections of the masseter myofiber areas between lean and obese dietary phenotypes treated with botulinum toxin type A [22]; however, the influence of obesity on masticatory muscle structure and function remained unclear.

The coordinated movement of the jaw, tongue, masticatory muscles, and temporomandibular joint performs mastication. The motor patterns underlying mastication are programmed by the central pattern generator (CPG) located in the brainstem [23,24]. The CPG receives afferent inputs from orofacial receptors [25] and higher brain centers, such as the cortical masticatory area (CMA) [26], thereby controlling the jaw movement neuromuscular behavior. The CMA is composed of two areas: the anterior area (i.e., A-area) located in the primary motor cortex and the posterior area (i.e., P-area) located in the dorsal part of the insular cortex [27]. Repetitive intracortical micro-stimulation in the A-area and P-area induces rhythmic jaw movements (RJMs) with electromyographic (EMG) activities of the masticatory muscles [28,29]. Coordinated rhythmic tongue movement and saliva secretion accompany CMA-induced RJMs, which strongly resemble natural mastication [29,30]. Moreover, the RJMs pattern is different if stimulated in different regions of the CMA [27,31]. 

Clinical studies recorded voluntary masticatory movements to assess masticatory behaviors. Voluntary masticatory movements, however, are affected by appetite [32], food form and flavor [33], etc.; therefore, assessing voluntary mastication movement is not a good way to investigate obesity’s effect on the coordinated motor function of masticatory components. Thus, in this study, we aimed to investigate obesity’s effect on cortically induced rhythmic jaw movements [34,35].

This study investigated the effect of obesity on cortically induced RJM patterns and neuromuscular behaviors of masticatory muscles during electrical stimulation in the A-area and P-area of OZRs and LZRs. In a well-established genetic obesity model [36], OZRs and their counterparts, LZRs, were used as the experimental and control groups, respectively. The microstructure of jaw movement trajectories was analyzed by size and time parameters to compare the RJM patterns. EMG activities were analyzed to evaluate the masticatory muscle functions. Our null hypothesis was that no differences in RJM patterns and masticatory EMG activities would exist between the OZRs and LZRs.

## 2. Materials and Methods

This study complied with the Animal Care Standards of Tokyo Medical and Dental University, according to the Institutional Animal Care and Use Committee (A2021-269) guidelines, and followed the ARRIVE guidelines.

### 2.1. Animal Model

This study followed a parallel control design. Male OZRs (fa/fa genotype) as the experimental group and their littermate LZRs (Fa/Fa or Fa/fa genotype) as the control group was purchased (7 each) from SLC (SLC Inc., Tokyo, Japan). They were housed (two per cage) starting from 7 weeks of age under a 12:12 h light/dark cycle with ad libitum access to food and water. The animals were fed a standard chow diet (CE-2; CLEA Inc., Tokyo, Japan). Body weight and food intake were measured at the age of 10 weeks. Additionally, food intake was measured for 3 days, and the average daily intake was calculated.

### 2.2. Surgical Preparation

Animals underwent the experimental procedure at 10 weeks of age following a protocol similar to that used in our previous studies [34,35]. Ketamine-HCl ((100 mg/kg, intraperitoneal (IP)) was administered before craniotomy and EMG electrode insertion. Supplementary doses were injected whenever necessary to maintain a constant level of anesthesia after assessing vibrissa movements, pinch-withdrawal, and corneal reflexes throughout the experiments. The local anesthetic lidocaine hydrochloride (2%) was injected subcutaneously below the planned surgical area. A thermoregulated heating pad maintained the body temperature at 37–38 °C.

**Figure 1:**
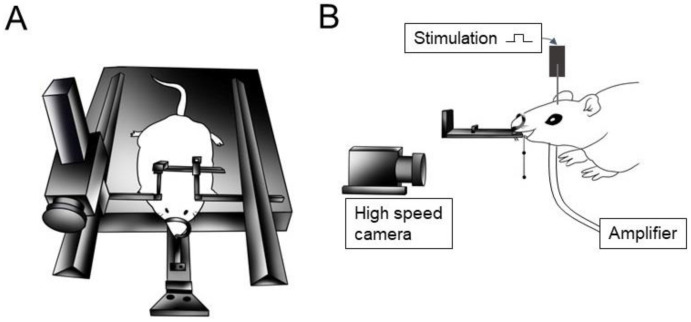
Experimental settings for cortically induced jaw movements. (**A**) Schematic drawing of the rat fixation. The heads of anesthetized rats are fixed to a stereotaxic frame. (**B**) Schematic drawing of the experimental setting. Rhythmical jaw movements were elicited by electrical stimulations in the anterior and posterior areas of the cortical masticatory area through a microelectrode. A high-speed digital camera was set directly before the marker to record rhythmical jaw movements. Electromyographic activity signals of the right anterior digastric and right masseter were filtered and amplified using a multichannel amplifier (for details, see the text).

A midline incision was made along the neck from the mandible on the ventral surface to the rostral portion to expose the right side of the masseter (i.e., jaw-closer) and anterior digastric (i.e., jaw-opener) muscles to record the EMG activity of the jaw muscles. Bipolar EMG electrodes (40 gauge, single-stranded, Teflon-insulated stainless-steel wires with a 2 mm interelectrode distance) were inserted into the muscles to record the EMG activity. Part of the left frontal and parietal bones was drilled using a dental bar to expose the outer surface of the masticatory cortex. The dura mater was covered with paraffin liquid oil (37 °C). The lower incisors were ground to approximately 1 mm to minimize interference of jaw and tongue movements. The rats were then secured in a stereotaxic apparatus (SN-2 and Sm-15 M; Narishige Scientific Instruments, Tokyo, Japan) (Figure 1). 

A fine glass-insulated tungsten microelectrode (shaft diameter, 100 µm; impedance 1–3 MΩ at 1 kHz; Unique Medical, Tokyo, Japan) was inserted vertically into the masticatory area of the left side of the cerebral cortex. Electrical stimulation of 0.5-ms duration, 20 Hz, and 120 µA for 8 s was applied to the left A-area (3–4 mm anterior, 2–3.5 mm lateral to bregma, 2–3.5 mm deep from the cortical surface), and electrical stimulation of 0.5 ms duration, 20 Hz, and 180 µA for 8 s was applied to the left P-area (0–2 mm rostral, 4–5.5 mm lateral to bregma, 4–5 mm ventral). A reference electrode was attached to the exposed neck muscle. RJMs were determined using mandibular movements and rhythmic bursts of the right anterior digastric (RAD) or masseter (RM) muscle EMG activities. Three trials were performed at each stimulation site.

### 2.3. Recording of Jaw Movement and EMG Activities

For recording sessions, a wire (0.7 mm in thickness) attached to a marker was placed between the lower incisors attached with dental resin. A digital high-speed HAS-U1M camera (DITEC T Corp, Tokyo, Japan) was set directly in front of the marker to detect jaw movement. During stimulation, the jaw movements were videotaped, and 2D motion analysis system software (Dipp-motion V, Ver1.1.31; DITECT Corp) was used to refine the marker position of the jaw movements. EMG activity signals were filtered and amplified using a multichannel amplifier (MEG-6108; 1000× gain, bandpass 0.3–3 kHz; Nihon Kohden, Tokyo, Japan), and all EMG waveforms were rectified and averaged. Data were analyzed offline using the CED 1401 interface and Spike2 software for Windows, version 5.21 (Cambridge Electronic Design, Cambridge, UK). The EMG responses of each muscle were analyzed using a fast sweep. The power spectrum was displayed using a fast Fourier transform to analyze the specific characteristics of EMG. The RJM and EMG activity recordings were stored on a computer disk. Jaw movement patterns during the stimulation were observed in the frontal plane. The mean of each parameter was measured for ten chewing cycles in the early, middle, and late stimulation periods, and the average was calculated. EMG bursts were identified when the rectified EMG exceeded the mean by two standard deviations (SDs).

### 2.4. Data Analyses of Jaw Movements and EMG Activities

Jaw movement patterns during the stimulation were observed in the frontal plane. The jaw movement parameters measured were (1) gape size (i.e., vertical excursion between maximum opening and maximum closing), (2) lateral excursion (i.e., the horizontal distance between minimum jaw-opening position and the most lateral jaw position), (3) vertical jaw-opening speed (i.e., rate of vertical jaw movement distance per second), (4) jaw-opening duration (i.e., the time between maximum closing and subsequent maximum opening), (5) jaw-closing duration (i.e., the time between maximum opening and subsequent maximum closing), (6) total cycle duration (i.e., duration between two consecutive maximum openings), and (7) changes in amplitude of vertical jaw movements (i.e., gape size). Lateral excursion, vertical jaw-opening speed, jaw-opening duration, closing duration, and cycle duration concerning the time in the sequence (i.e., total duration of stimulation) are divided into three equal periods—early, middle, and late—corresponding to the first, second, and third periods, for 2 s, respectively. The mean data values for each parameter were measured using ten chewing cycles in each period. The average values during the entire experimental task were measured as the mean of each period.

EMG activity of the RAD muscle was analyzed regarding (1) onset latency (i.e., the interval between the onset of the stimulus and the onset of the first response), (2) peak-to-peak amplitude (i.e., amplitude analyzed by the peak–peak function of Spike 2 in data not rectified), (3) duration (i.e., the interval between the onset and offset of RAD EMG activity), (4) median frequency of the power spectrum (i.e., the EMG power spectrum divided into two halves with equal amplitudes), and (5) mean frequency of the power spectrum (i.e., average frequency, which is calculated as the sum of the product of the EMG power spectrum and the frequency divided by the total sum of the power spectrum). EMG bursts were identified when the rectified EMG exceeded the mean by 2 SDs. All values are expressed as the mean ± standard deviation (SD).

### 2.5. Histological Identification of Electrode Positioning

**Figure 2:**
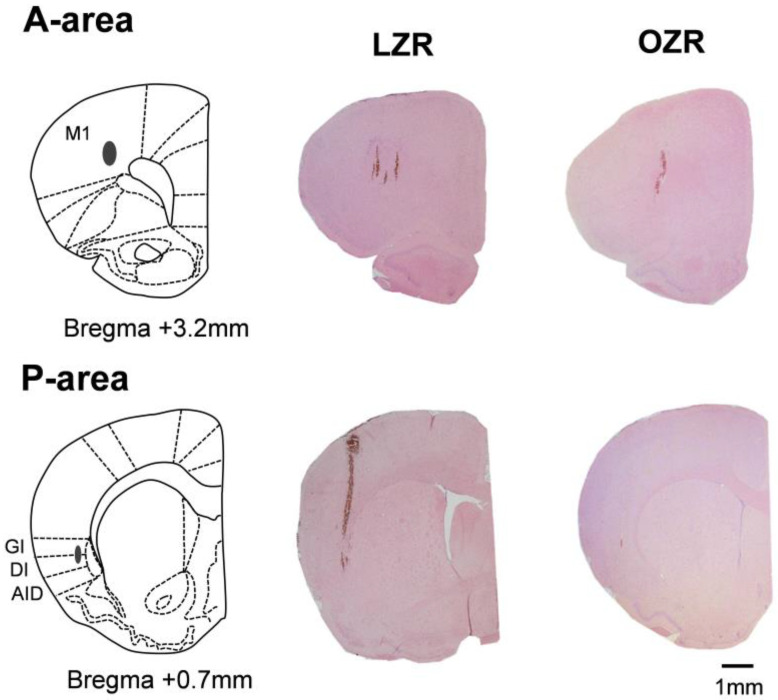
Hematoxylin–eosin-stained coronal sections (6 µm) with a schematic drawing of the stimulation sites in the anterior and posterolateral parts of the left cortical masticatory area. Gray circles indicate the electrolytic lesions of the stimulation sites. Relative distances from the bregma in the rostral (0.7 and 3.2 mm anterior to the bregma) direction are depicted. The schematic drawings were made according to the rat brain in stereotaxic coordinates [37], showing the histological composition of the left brain and the location of stimulation sites. Scale bar, 1 mm. Abbreviations: M1, primary motor cortex; GI, granular insular cortex; DI, dysgranular insular cortex; and AID, dorsal part of the agranular insular cortex. A-area, anterior area; P-area, posterior area.

The rats remained in the stereotaxic apparatus after the experiment was completed. First, they were anesthetized with ketamine-HCl (20 mg/kg, IP). Electrical lesions were created by passing currents (30 µA for 20 s) through the stimulating electrode in the same area to check the location of the electrode tips. The rats were then deeply anesthetized and perfused with 100 mL of phosphate-buffered saline (PBS; pH 7.4) through the left cardiac ventricle, followed by 300 mL of a fixative solution of 4% paraformaldehyde. Serial brain coronal sections (6 µm in thickness) were cut and counterstained with hematoxylin and eosin stain. The locations of the electrode tips were confirmed under a light microscope and verified using a reference (Figure 2).

### 2.6. Statistical Analyses

All data are expressed as mean ± SD. The Shapiro–Wilk test was used to determine whether the variables followed a normal distribution. The unpaired *t*-test was used for comparisons between OZRs and LZRs. Two-way analysis of variance (ANOVA) was used for intergroup and intragroup comparisons. Statistical analysis was performed using SPSS for Windows, version 23 (SPSS, Inc., Chicago, IL, USA). Statistical significance was set at *p* < 0.05.

## 3. Results

### 3.1. Body Weight and Food Intake

**Figure 3:**
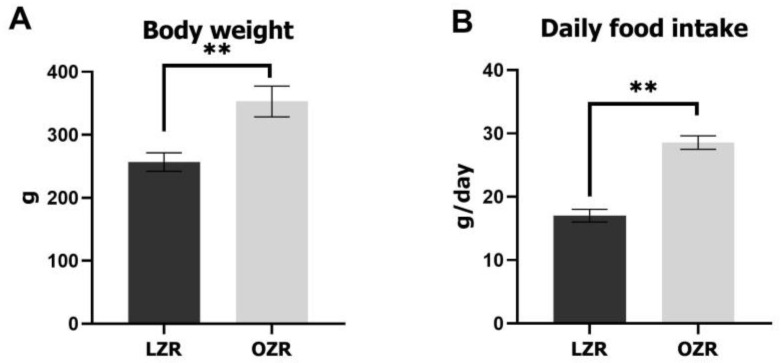
Comparison of (**A**) body weight and (**B**) daily food intake between lean and obese Zucker rats. Error bars indicate the means ± SD. Asterisks (** *p* < 0.01) denote significant differences between LZRs and OZRs. Abbreviations: LZR, lean Zucker rat; OZR, obese Zucker rat; SD, standard deviation.

The body weight and food intake of the rats were measured at the age of 10 weeks. The results revealed that the body weight and food intake were significantly larger in OZRs than in LZRs (Figure 3).

### 3.2. RJMs Elicited by Stimulation of the A-Area and P-Area of the CMA

**Figure 4:**
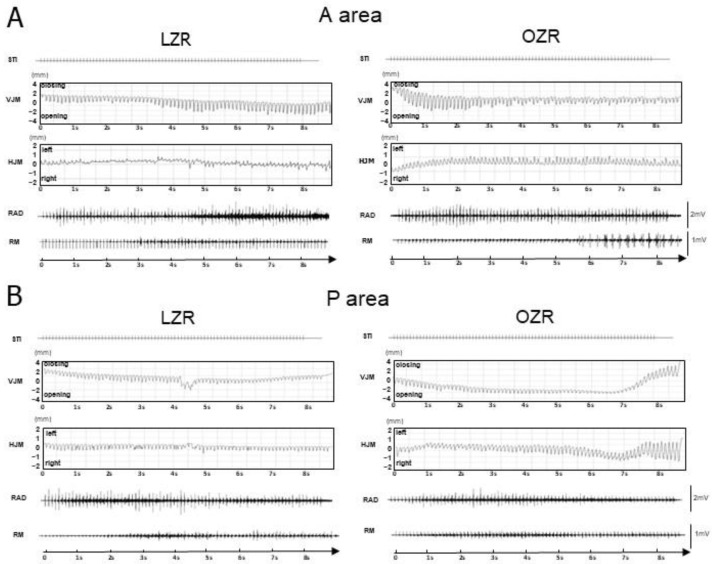
A typical example of jaw movement patterns and electromyographic activity induced by electrical stimulation of the cortical masticatory area. Stimulating the (**A**) A-area and (**B**) P-area in LZRs and OZRs evokes EMG activity. Abbreviations: EMG, electromyographic; A-area, anterior area; P-area, posterior area; LZR, lean Zucker rat; OZR, obese Zucker rat; STI, stimulation; VJM, vertical jaw movements; HJM, horizontal jaw movements; RM, right masseter; RAD, right anterior digastric.

Typical jaw movement patterns and EMG activity during stimulation in the A-area and P-area are shown in Figure 4. The jaw position was stable at rest, and the distance between the upper and lower incisors was approximately 2–2.5 mm before stimulation. We observed RJMs with alternating activities of the RAD and RM muscles during low-frequency (10 Hz) long-train stimulation in the A-area and P-area. The EMG activity of the RAD muscle was time-locked to each stimulus pulse during the stimulation. In contrast, the masseter EMG activity was absent in the early period and started to show bursts during the middle period of stimulation when the jaw was fully opened. Our study only analyzed the right anterior muscle EMG activity because RM activity was weak and unevenly distributed during stimulation. We found a vertical pattern of RJMs regarding the RJM pattern, which involved jaw-opening and -closing movements with a rightward lateral shift during both A-area and P-area stimulations.

**Figure 5:**
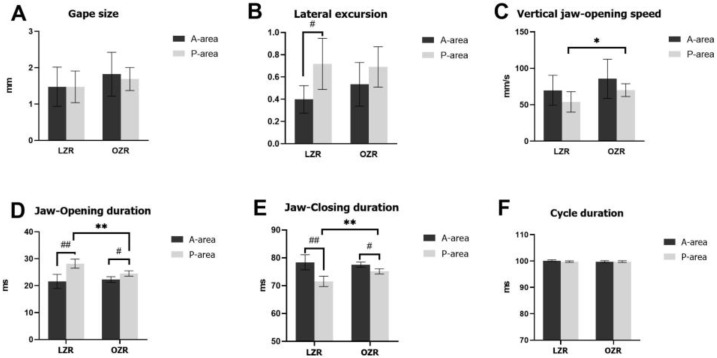
The effect of obesity on RJM patterns during A-area and P-area stimulations. (**A**) Gape size, (**B**) lateral excursion, (**C**) the speed of vertical jaw movements, (**D**) jaw-opening duration, (**E**) jaw-closing duration, (**F**) and total cycle duration. Error bars indicate the means ± SD. Asterisks (* *p* < 0.05 and ** *p* < 0.01) denote significant differences between LZRs and OZRs. Hashes (# *p* < 0.05 and ## *p* < 0.01) denote significant differences between A-area and P-area elicited jaw movements within the same group. Abbreviations: A-area, anterior area; P-area, posterior area; LZR, lean Zucker rat; OZR, obese Zucker rat; SD, standard deviation.

Jaw movements were recorded and analyzed during the sustained stimulation. The results of the parameters measuring RJM patterns are shown in Figure 5. The gape size and lateral excursion represent the vertical and lateral opening capacity, respectively, whereas the speed of vertical jaw movement reflects the jaw-opening muscle’s contractile ability [38,39]. There was no significant intergroup/intragroup difference in the gape size. Regarding lateral excursion, there is no difference between OZRs and LZRs; However, the intragroup comparison revealed that P-area-stimulated RJMs had higher (*p* < 0.05) lateral excursion than A-area-stimulated RJMs in the LZRs group, indicating different microstructure patterns of RJMs elicited by two areas of the cortical masticatory area. As for the vertical jaw-opening speed, OZRs showed a significantly higher (*p* < 0.05) vertical jaw-opening speed than LZRs during P-area stimulation; however, there was no significant difference during A-area stimulation.

The duration of jaw movements represents the rhythmic pattern of jaw movement microstructure. Compared to LZRs, OZRs showed a significantly shorter (*p* < 0.05) jaw-opening duration and a significantly longer (*p* < 0.05) jaw-closing duration during P-area stimulations; however, no differences were observed during A-area simulations between the two groups. Moreover, the cycle duration was steady during the experiment and showed no significant difference between OZRs and LZRs during A-area and P-area stimulations. The intragroup comparison revealed that P-area-stimulated RJMs had longer (*p* < 0.05) jaw-opening duration and shorter jaw-closing duration (*p* < 0.05) than A-area-stimulated RJMs in both OZRs and LZRs groups. 

The early, middle, and late periods of vertical jaw movements were measured to determine time-dependent changes in jaw movement sequences. A two-way ANOVA was conducted to examine the effect of time in sequence and obesity on the RJM parameters. (Table 1). Consistent with the results of the average parameters of the entire experimental task described above, obesity has a significant effect on the opening duration (*p* < 0.0001), closing duration (*p* < 0.0001), and vertical jaw-opening speed (*p* < 0.05) only during P-area stimulation; however, obesity does not affect gape size, lateral excursion, or cycle duration.

A simple main effects analysis showed that time sequence among the early, middle, and late periods had no significant effect on the parameters describing RJM patterns except for jaw-opening duration (*p* < 0.05) elicited by A-area stimulation. The result showed that the RJM patterns stayed stable during the 8 s stimulations in both A-area and P-area.

### 3.3. EMG Activities Induced by Stimulations of the A-and P-Area of the CMA 

**Figure 6:**
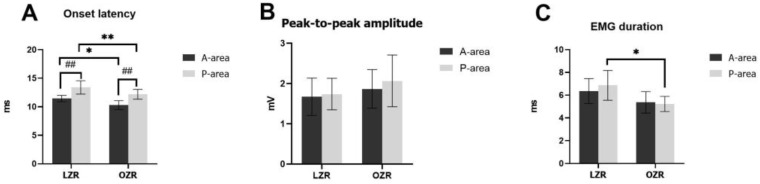
The electromyographic activity of the right anterior digastric muscle was evoked by electrical stimulation in the A-area and P-area. (**A**) Onset latency and (**B**) peak-to-peak amplitude and (**C**) duration of LZR and OZR. Error bars indicate the means ± SD. Asterisks (* *p* < 0.05 and ** *p* < 0.01) denote significant differences between LZRs and OZRs. Hashes (## *p* < 0.01) denote significant differences between A-area- and P-area-elicited jaw movements within the same group. Abbreviations: A-area, anterior area; P-area, posterior area; LZR, lean Zucker rat; OZR, obese Zucker rat; SD, standard deviation.

The EMG activities in the RAD and RM muscles were recorded during both A-area and P-area stimulations while the stimulation was sustained. The EMG onset latency, peak-to-peak amplitude and durations are shown in Figure 6. Compared to LZRs, the onset latency was significantly shorter (*p* < 0.05) in OZRs during both A-area and P-area stimulations, showing that the time gap between electrical stimulation and the start of muscle activity was shorter in OZRs than that in LZRs. There was no significant difference in the peak-to-peak amplitude between the OZR and LZR groups during both A-area and P-area stimulations. The RAD EMG duration was significantly shorter in OZRs than in LZRs during P-area stimulation, indicating a shorter RAD contraction time in OZRs; however, two groups had no significant difference in RAD durations during A-area stimulation. The intragroup comparison showed that the RAD EMG onset latency was significantly longer (*p* < 0.05) in P-area stimulation than in A-area stimulation in both OZRs and LZRs.

**Figure 7:**
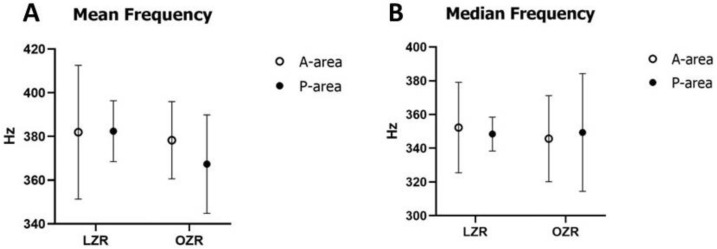
(**A**) Mean and (**B**) Median frequencies of the anterior digastric muscle during stimulation in the A and P parts of the cortical masticatory area. Error bars indicate the means ± SD. Abbreviations: A-area, anterior area; P-area, posterior area; LZR, lean Zucker rat; OZR, obese Zucker rat; SD, standard deviation.

The average mean frequency and median frequency were not different between two groups during both A-area and P-area stimulations in the power spectrum analysis of the EMG signal (Figure 7).

## 4. Discussion

This study demonstrated that the P-area-induced RJM patterns and RAD EMG activities (duration) were affected by obesity; therefore, the null hypothesis was rejected, and obesity impacts the coordinated motor function of masticatory components. The P-area elicited a more lateral-shifting and slower-opening RJM pattern than the A-area. OZRs exhibited a faster-opening RJM pattern with shorter digastric muscle contraction time compared to LZRs during P-area stimulation regarding obesity’s effect on cortically induced RJMs. We speculate that other masticatory components, such as tongue movement, participated in the obesity-related dynamic changes of coordinated movement of masticatory components and mutually affected each other’s contraction time, which corresponds to the findings of shorter contraction time of digastric muscle. Nonetheless, findings of our study are important because they might give insight into clinical findings about an association between obesity and masticatory movement.

### 4.1. Obesity and Low-Satiety Responsiveness in OZRs at the Age of 10 Weeks

Obese Zucker rats (fa/fa), a popular genetic obesity mode, become obese because of increased food intake secondary to a genetic leptin receptor deficiency, which results in a low-satiety response [40]. OZRs have a greater daily food intake and higher body weight than LZRs at age 5–20 weeks [41]. In agreement with previous studies, the body weight and daily food intake were significantly higher in OZRs than in LZRs in our study, reflecting the genetically obese state with low-satiety responsiveness in OZRs. 

In our previous studies, the microstructure patterns of RJMs, including gape size and lateral excursion, elicited by the A-area and P-area, were fully developed in rats at 9 weeks of age [34,35]. The response properties of rat periodontal mechanoreceptors and related neurons mature within 5 weeks during craniofacial maturation [42], whereas mandibular growth reaches a plateau in rats after 9 weeks of age [43]. Moreover, the number and size of skeletal muscle fibers in rats undergo intense growth from 3 to 10 weeks of age and attain stable growth after 10 weeks [44]; therefore, we conducted the experiments using 10-week-old rats to assess differences in jaw movement and muscle EMG activities between the OZRs and LZRs.

### 4.2. Effects of Stimulation Sites on Cortically Induced RJMs

We found a vertical pattern of jaw-opening and -closing movements in RJMs that shifted to the right during stimulation of A-area and P-area. P-area-stimulated RJMs exhibited larger lateral excursions in LZRs group, reflecting a more lateral shift pattern than A-area stimulated RJMs. In agreement with this study, previous studies have reported that A-area stimulation induces licking-like behavior, which consists of simple jaw-opening/closing movements; however, P-area stimulation induces masticatory-like behavior, which consists of complex lateral and forward jaw-opening/closing movements with vigorous saliva secretion in rats [27,30,34,35].

The gape size did not differ between A-area- and P-area-elicited RJMs. As the threshold of cortically elicited RJMs is 50–200 μA in the A-area and 50–220 μA in the P-area [27,34,35], we believed that the current of 120 μA in the A-area and 180 μA in the P-area used in this experiment were enough to induce active activity in both CMA areas. The stimulations in the A- and P-areas activated digastric muscle activity. The same gape size observed between the two stimulated areas shows that A-area and P-area can activate enough digastric muscle activity to open the jaw fully. 

One of the novel findings from our study is that P-area-stimulated RJMs showed longer vertical jaw-opening duration and shorter jaw-closing duration than A-area-stimulated RJMs in both the LZRs and OZRs groups, indicating that the two typical RJMs were not only spatially but also temporally different during an opening-closing cycle. Interestingly, the digastric muscle, the main jaw-opening muscle, did not show any difference in contraction force and contraction time indicators in EMG data. Thus, we speculate that the duration difference between A-area- and P-area-stimulated RJMs was because of reasons other than digastric muscle. 

One of the reasons might be the different tongue movements between the two stimulated areas. Coordination of tongue movement during cortically induced RJMs was observed in rabbits [31,45] and guinea pigs [46], and it has been reported that each type of jaw movement was associated with a particular pattern of tongue and hyoid movements [31]. A previous study reported that cortically induced RJMs were influenced by tongue protrusion elicited by electrical stimulation on the hypoglossal (XII) nerve in rats [47], indicating a tight connection between tongue and jaw movement patterns. A superior-laryngeal-nerve-elicited swallowing reflex, involving tongue movement is inhibited by A-area stimulation, but not P-area stimulation [48], showing different functions of the two masticatory areas involved in tongue control. Whether the different tongue movements are the main reason for the different RJM patterns during A-area and P-area stimulation still needs further investigation.

Recent studies have also reported that P-area stimulation, but not A-area stimulation, can evoke saliva secretion during RJMs [30]. We did not measure saliva secretion in our study; therefore, whether the amount and speed of saliva secretion were associated with different jaw movement microstructure needs further investigation.

The onset latency of RAD in EMG data was significantly longer in P-area-elicited activities than in A-area-elicited activities, showing different corticofugal projections from the A-area and P-area to CPG. The A-area is located in the primary motor cortex, and the P-area is located in the dorsal part of the insular cortex. The reciprocal connection between the A-area and the P-area is weak. They appear to work independently of each other because ablation of the A-area or P-area did not affect the pattern of RJMs evoked by the stimulation of the other area [30]. 

### 4.3. Obesity-Related Changes in RJMs Elicited by Stimulations of the A-Area and P-Area

In this study, gape size and lateral excursion did not differ between OZRs and LZRs in A-area- and P-area-elicited RJMs. Previous studies have reported that the maximum jaw movement capacity was positively correlated with variations in mandibular and condylar growth [49]. Similarly, lateral movements were associated with the shape of the temporomandibular joint, which would restrict lateral movements [34]. The severe obesity state caused by a high-fat diet affects the mandibular condyle bone quality by decreasing mineralization in mice regarding the obesity impact on the temporomandibular joint [50]. Moreover, obesity is a risk factor for temporomandibular disorders [51]. Our study’s unchanged gape size and lateral excursion indicated a similar condyle and temporomandibular joint morphology between OZRs and LZRs at 10 weeks.

We observed significantly shorter jaw-opening durations in OZRs than in LZRs during P-area stimulation. This result is consistent with the EMG findings that the RAD EMG duration was significantly shorter in OZRs than in LZRs during P-area stimulation. Despite the shortened jaw-opening time, the jaw still opened to full mouth, and the vertical jaw-opening speed appeared to be higher in OZRs than in LZRs during P-area stimulation. Previous studies have reported a positive relationship between muscle contraction force and jaw movement speed [38,39]; however, our study found no difference in RAD EMG amplitude, an indicator of muscle contraction output, between the two groups. Therefore, other masticatory components might have contributed to the higher jaw-opening speed and shorter jaw-opening duration. 

It is important to note that obesity influences various intraoral soft tissues to explain this result. For example, obesity is associated with more fat infiltration [11,12] and bigger muscle fiber size [11] in the tongue muscle. The fat infiltration mainly focuses on the anterior one-third of the tongue and might affect tongue stiffness [11]. Moreover, obesity is associated with upper air way soft tissue changes. Previous studies have reported upper airway narrowing [52] and pharyngeal collapsibility [53] in OZRs. 

Additionally, clinical studies have reported lateral pharyngeal wall enlargement and posterior tongue displacement in OSA patients [54], for which obesity is an important risk factor [15]. RJMs are the coordinated movement of the jaw, tongue, masticatory muscles, and temporomandibular joint. The tongue may retract to a lower position in OZRs than in LZRs because of obesity-related changes to intraoral structures, thus making dynamic changes to the coordinated movement of other masticatory components, such as the digastric muscle, and the shorter RAD contraction time is proof for this speculation. Only P-area stimulation showed obesity-related changes in cortically induced RJM patterns because the A-area and P-area might have evoked different tongue movements. The tongue protruded during A-area stimulation [55] and had a more complicated movement during P-area stimulation; however, further experiments on tongue muscle movement during cortical stimulation are needed to give direct proof to this speculation. 

Interestingly, obesity is not only associated with specific voluntary masticatory patterns [16,17,18,19,20], but also connected with changed P-area-stimulated RJM patterns. Voluntary and cortically induced jaw movement are programmed by the CPG [23,24] and performed by masticatory components, such as digastric muscles, tongue, and temporomandibular joint; therefore, the mechanism underlying the obesity-related dynamic change of coordinated movement of masticatory components might also contribute to obese eating behaviors, such as faster eating. Moreover, our study has a great advantage over traditional clinical studies on voluntary mastication because we investigated cortically induced RJMs in anesthesia rats to avoid the impact of central appetite and food flavor, which affect voluntary mastication. Additionally, obesity is an important risk factor for snoring and obstructive sleep apnea [15]. The possible tongue movement change underlying the mechanism of our findings may also be supportive evidence of the relationship between obesity and obstructive sleep apnea.

### 4.4. Obesity-Related Changes in the Neuromuscular Function of the RAD Muscle

In our study, analysis of the EMG activity showed that the onset latency of the RAD muscle in OZRs was significantly shorter than that in LZRs during both A-area and P-area stimulations. Onset latency is associated with conduction velocity of nerve fibers between the brain and RAD [56]; therefore, our results showed that OZRs have higher nerve conduction velocity (NCV) than LZRs. It has been reported that the NCV is positively correlated with BMI in the motor ulnar nerve [57,58,59]. Body temperature is one of the most important factors affecting NCV [60,61]. During our study, although the temperature of the heating pad remained the same in every experiment, the perineural temperature might be higher in OZRs because of excess subcutaneous fat that acted as thermal insulation. Moreover, it has been shown that the correlation between NCV and BMI in previous studies and our study might be related to the extent of epineural fat, which acts as insulator for the nerve and thus maintains the temperature [62]. Muscle fiber conduction velocity (MFCV) of the RAD muscle might also contribute partly to the onset latency. The OZRs might have higher MFCV because the better thermal insulation formed by excess subcutaneous fat may result in higher muscle temperature, which plays an important role in modulating MFCV [63]. 

The peak-to-peak amplitude is mainly affected by muscle diameter and the number of recruited motor units [64,65] closest to the electrode. We used the peak-to-peak amplitude as a force indicator because it is positively correlated with force value [66]; therefore, the results, in which there is no difference in peak-to-peak amplitude between OZRs and LZRs, showed that the maximal contraction force did not differ between the two groups. There are two opposing trends in how obesity affects skeletal muscle contractile properties. Firstly, obesity is associated with greater absolute force in anti-gravity muscles [67]. Contrarily, obesity can decrease muscular power output by altering fiber type composition and disrupting calcium cycling [9,68]. Calcium signaling plays an important role in facilitating muscle contraction and relaxation [69,70] and signaling changes to muscle phenotype [71]. Obesity disrupts calcium signaling by inducing calcium leaks from the sarcoplasmic reticulum through excess production of reactive oxygen species [8]. Moreover, the increased insulin levels in obesity inhibit AMPK activity in muscle, thereby suppressing slow-fiber-type expression [72]. The digastric muscle is not an antigravity muscle, and we speculate that the obesity-related microscopic effects on digastric muscle have not yet lead to observable force reduction at the age of young adults (i.e., 10 w) in OZRs.

Previous studies generally measured the mean and median frequencies to monitor muscle fatigue [73]. The mean frequency is calculated as the sum of the product of the EMG power spectrum and the frequency divided by the total sum of the power spectrum. Contrarily, the median frequency is the frequency at which the EMG power spectrum is divided into two regions with equal amplitude [74]. This study calculated the mean and median frequencies following power-spectral analysis. Muscle geometry, including fiber diameter, muscle length, and fiber type, significantly affects the time-varying EMG spectrum. A previous study reported a positive relationship between frequency in the EMG spectrogram and muscle fiber size and muscle recruitment [75]. Another study has revealed that muscles with a greater percentage of fast fibers and a larger cross-sectional area showed a higher mean frequency in EMG data [76]. A similar correlation has been reported between the proportions of different fiber types and the mean frequency of EMG activities [77]. In our study, the mean and median frequencies of RAD muscle activities were not different in OZRs than in LZRs during both A-area and P-area stimulations, reflecting a similar muscle fiber size and fiber composition between the two groups.

### 4.5. Limitations

In this study, we found that obesity influences cortically induced RJM patterns. The OZRs showed a higher jaw-opening speed but unchanged digastric muscle force, which shows the participation of other masticatory components in the mechanism. We speculate that tongue movement serves a key role in obesity-related dynamic changes of the coordinated movements of masticatory components because obesity affects intraoral tissue structure, and the shorter digastric muscle contraction time is a part of the coordinated masticatory movement change. Although we made this speculation based on the findings of previous studies and our study, further research about obesity’s effect on tongue movement is needed to give direct evidence. Moreover, cortically induced RJMs include other masticatory components, such as saliva secretion; therefore, whether obesity affects RJM patterns by influencing saliva secretion needs further investigation. Additionally, obesity-related microscopic effects alter muscle fiber type and muscle contraction ability; therefore, we warrant further studies on masticatory muscle type and signaling processes affected by obesity.

## 5. Conclusions

This study investigated the effect of obesity on the coordinated movement of masticatory components by assessing cortically induced RJM patterns and masticatory muscle EMG activities. This study revealed the following: (1) A-area and P-area elicit different jaw movement patterns, reflecting the different functions of the A- and P-areas. (2) A similar vertical and lateral-opening capacity between OZRs and LZRs during A-area and P-area stimulations was found. (3) OZRs exhibited cortically induced RJM patterns with a shorter opening duration and faster opening speed than LZRs. The unchanged digastric muscle force parameters, however, showed the participation of other masticatory components in the mechanism. We speculated that not just one muscle but the coordination of most masticatory muscle movements is changed in OZRs. Tongue movement plays a crucial role in modulating the dynamic changes of coordinated movement of masticatory components, which include shorter RAD contraction time in our results. (4) The onset latency of RAD was significantly shorter in OZRs than that in LZRs, showing a higher perineural temperature because of excess of fat. (5) EMG amplitude and frequency parameters of the RAD muscle were not different, showing a similar RAD muscle contraction ability and muscle geometry between the two groups. These findings show that although obesity has a limited effect on digastric muscle contractile ability and muscle geometry, and that digastric muscle still serves as a part of altered coordinated movement of masticatory components by shorter contraction time. We speculated that tongue movement affected by obesity-related organic changes to intraoral tissues might be the main factor causing the coordinated movement change of masticatory changes. Further investigation into this mechanism is warranted because it might provide insights into obesity-related clinical problems with masticatory muscle movement.

## Figures and Tables

**Table 1:** Variations of parameters describing cortically induced RJM patterns with time in the sequence. The total duration of the stimulation sequence was divided into three equal periods (2.5 s): early, middle, and late periods. A two-way ANOVA was conducted to examine the effect of time in sequence and obesity on the parameters that describe RJM patterns. Abbreviations: A-area, anterior area; P-area, posterior area; LZR, lean Zucker rat; OZR, obese Zucker rat; RJM, rhythmic jaw movement.

	LZRs						OZRs						Two-Way ANOVA		
	Early		Middle		Late		Early		Middle		Late		Effect (*p* Value)		Interaction
	Mean	SD	Mean	SD	Mean	SD	Mean	SD	Mean	SD	Mean	SD	Time	Obesity	*p* Value
								A-area							
Gape size (mm)	1.8	0.6	1.2	0.4	1.4	0.6	2.0	0.4	1.8	0.7	1.5	0.6	NS	NS	NS
Lateral excursion (mm)	0.4	0.1	0.5	0.1	0.5	0.1	0.5	0.1	0.5	0.2	0.5	0.1	NS	NS	NS
Vertical jaw-opening speed (mm/s)	83.2	16.4	55.7	22.9	67.2	36.6	90.6	15.2	85.5	32.0	74.5	38.0	NS	NS	NS
Jaw-opening duration (ms)	22.3	3.2	20.7	2.0	21.9	2.6	23.5	1.3	21.5	1.5	21.9	1.3	0.01	NS	NS
Jaw-closing duration (ms)	77.5	3.2	79.3	2.1	78.3	2.7	76.4	1.1	78.1	1.4	78.6	1.2	NS	NS	NS
Cycle duration (ms)	99.8	0.3	99.9	0.5	100.1	0.7	99.8	0.7	99.6	0.3	99.7	0.5	NS	NS	NS
								P-area							
Gape size (mm)	1.3	0.4	1.3	0.6	1.6	0.9	1.5	0.3	1.4	0.3	1.7	0.5	NS	NS	NS
Lateral excursion (mm)	0.7	0.2	0.7	0.2	0.7	0.2	0.7	0.2	0.7	0.2	0.6	0.2	NS	NS	NS
Vertical jaw-opening speed (mm/s)	48.9	14.5	48.6	0.2	54.9	23.1	65.2	19.9	64.9	15.2	72.5	20.7	NS	0.02	NS
Jaw-opening duration (ms)	28.9	2.1	27.1	0.2	28.0	1.9	24.1	1.2	24.4	1.1	24.5	1.3	NS	<0.0001	NS
Jaw-closing duration (ms)	70.6	2.1	72.8	0.2	72.1	2.0	75.7	1.2	75.6	0.9	75.3	1.3	NS	<0.0001	NS
Cycle duration (ms)	70.6	0.8	99.9	0.2	99.9	0.3	99.8	0.4	99.9	0.4	99.8	0.5	NS	NS	NS
Two-way ANOVA: NS, not significant; SD, standard deviation.															

## Data Availability

The authors will, without undue reservation, make the raw data supporting the conclusions of this article available to any qualified researcher.
